# Genome Sequence of the Moderately Acidophilic Sulfate-Reducing Firmicute *Desulfosporosinus acididurans* (Strain M1^T^)

**DOI:** 10.1128/genomeA.00881-15

**Published:** 2015-08-06

**Authors:** Patrick Petzsch, Anja Poehlein, D. Barrie Johnson, Rolf Daniel, Michael Schlömann, Martin Mühling

**Affiliations:** a Institute of Biological Sciences, TU Bergakademie Freiberg, Freiberg, Germany; b Genomic and Applied Microbiology & Göttingen Genomics Laboratory, Georg-August-Universität Göttingen, Göttingen, Germany; c College of Natural Sciences, Bangor University, Bangor, United Kingdom

## Abstract

Microbial dissimilatory sulfate reduction is commonplace in many anaerobic environments, though few acidophilic bacteria are known to mediate this process. We report the 4.64-Mb draft genome of the type strain of the moderate acidophile *Desulfosporosinus acididurans*, which was isolated from acidic sediment in a river draining the Soufrière volcano, Montserrat.

## GENOME ANNOUNCEMENT

Dissimilatory sulfate reduction mediated by microorganisms at low environmental pH has been observed for almost 50 years (1), though only very few acidophilic sulfate-reducing bacteria (SRB) have so far been isolated (e.g., references 1–4). Here, we report the draft genome sequence of strain M1^T^ (DSM 27692 and JCM 19471), the type strain of the novel species *Desulfosporosinus acididurans* (5). *D. acididurans* is a moderate acidophile that grows optimally at pH 5.5. It was isolated from sediment in the White River, Montserrat (West Indies), which drains the Soufrière volcano, several months before the cataclysmic eruption in 1995.

Chromosomal DNA of strain M1^T^ was isolated using the MasterPure complete DNA purification kit (Epicentre) and submitted to genome sequencing via a combined approach using both the Titanium chemistry of the 454 GS-FLX pyrosequencing system (Roche Life Sciences) and the Genome Analyzer IIx (2- × 112-bp paired-end sequencing; Illumina). Shotgun libraries were prepared according to the manufacturers’ protocols. Sequencing resulted in 3,244,857 reads from Illumina and in 83,381 reads from 454 sequencing. The *de novo* assembly performed with the Roche Newbler v2.9 and MIRA v3.4 assembler (6) resulted in 47 contigs. The average genome coverage is 10.65 (pyrosequencing) and 78.3 (Illumina). The genome of strain M1^T^ comprises 4.64 Mb and a G+C content of 41.79 mol%. Automated gene prediction was performed using Prodigal (7). Identification of rRNA and tRNA genes was achieved using RNAmmer (8) and tRNAscan (9), respectively. The Integrated Microbial Genomes–Expert Review (IMG-ER) system (10) was used for automated annotation, which was subsequently manually curated using the Swiss-Prot, TrEMBL, and InterPro databases (11). We identified 28 rRNA genes, 68 tRNA genes, and 4,393 protein-coding genes, from which 3,419 were assigned to putative functions.

The analysis of the genome sequence of *D. acididurans* indicates that it can utilize both inorganic phosphate and phosphonates as P sources and a variety of nitrogen sources. The 15 genes related to amino acid ABC-type transporters and 6 genes coding for di- or oligopeptide transporters may be related to the ability of strain M1^T^ to grow on yeast extract (5). Strain M1^T^ appears to fix dinitrogen or use nitrite, nitrate and urea as alternative N sources. Besides providing nitrogen, urea may also play a role in the adaptation to low pH (12, 13). The genome analysis further corroborates the experimental finding that sulfate, thiosulfate, sulfur (as polysulfide), nitrate, and ferric iron are used as electron acceptors (5). However, no gene encoding a dissimilatory ferric iron reductase was detected, which may be explained by currently limited genetic information on dissimilatory ferric iron reductases or point at an indirectly mediated reduction of ferric iron, for instance via concomitantly produced hydrogen sulfide. *D. acididurans* encodes the genes for CO_2_ fixation via the reductive acetyl coenzyme A (acetyl-CoA) pathway, which is used for autotrophic growth with H_2_/CO_2_ and sulfate as the electron acceptor (5).

### Nucleotide sequence accession numbers.

The results from this genome sequencing project have been deposited at GenBank under the accession number LDZY00000000. The version described in this paper is version LDZY00000000.1.
